# Accessory Gene Regulator-1 Locus Is Essential for Virulence and Pathogenesis of *Clostridium difficile*

**DOI:** 10.1128/mBio.01237-16

**Published:** 2016-08-16

**Authors:** Charles Darkoh, Chioma Odo, Herbert L. DuPont

**Affiliations:** a Department of Epidemiology, Human Genetics, and Environmental Sciences, Center For Infectious Diseases, University of Texas Health Science Center, School of Public Health, Houston, Texas, USA; b Microbiology and Molecular Genetics Program, University of Texas Graduate School of Biomedical Sciences, Houston, Texas, USA

## Abstract

*Clostridium difficile* infection (CDI) is responsible for most of the definable cases of antibiotic- and hospital-associated diarrhea worldwide and is a frequent cause of morbidity and mortality in older patients. *C. difficile*, a multidrug-resistant anaerobic pathogen, causes disease by producing toxins A and B, which are controlled by an accessory gene regulator (Agr) quorum signaling system. Some *C. difficile* strains encode two Agr loci in their genomes, designated *agr1* and *agr2*. The *agr1* locus is present in all of the *C. difficile* strains sequenced to date, whereas the *agr2* locus is present in a few strains. The functional roles of *agr1* and *agr2* in *C. difficile* toxin regulation and pathogenesis were unknown until now. Using allelic exchange, we deleted components of both *agr* loci and examined the mutants for toxin production and virulence. The results showed that the *agr1* mutant cannot produce toxins A and B; toxin production can be restored by complementation with wild-type *agr1*. Furthermore, the *agr1* mutant is able to colonize but unable to cause disease in a murine CDI model. These findings have profound implications for CDI treatment because we have uncovered a promising therapeutic target for the development of nonantibiotic drugs to treat this life-threatening emerging pathogen by targeting the toxins directly responsible for disease.

## IMPORTANCE

Within the last decade, the number of cases of *C. difficile* infections has been increasing exponentially in the United States, resulting in about 4.8 billion U.S. dollars in health care costs annually. As a multidrug-resistant, spore-forming, anaerobic pathogen, *C. difficile* overpopulates the colon after the gut microbiota has been altered by antibiotic therapy. With increasing resistance to antibiotic treatment of *C. difficile* infections, patients are experiencing higher costs of health care and a lower quality of life as treatment options decrease. During infection, *C. difficile* produces toxins A and B, which directly cause disease. As a result, the toxins have become promising nonantibiotic treatment targets. Here, we have identified a pathway responsible for activating the production of the toxins. This important finding opens up a unique therapeutic target for the development of a novel nonantibiotic therapy for *C. difficile* infections.

## INTRODUCTION

*Clostridium difficile* infections (CDIs) account for most of the cases of antibiotic- and hospital-associated diarrhea with defined etiology worldwide and are a frequent cause of morbidity and mortality in older hospitalized patients (1). CDI has traditionally been treated with broad-spectrum antibiotics. However, antibiotic therapy is the highest risk factor for CDI (2, 3), because it significantly alters and weakens the colonization resistance provided by the diverse microbiota that protects the colon. This allows multidrug-resistant *C. difficile*, usually a normal commensal, to proliferate and cause disease. Antibiotic-based treatment of CDI also leads to recurrence in as many as 25 to 30% of the cases (4, 5). Thus, there is a critical need for nonantibiotic CDI therapies that preserve the colonic microbiota, as either stand-alone or adjunctive therapies to augment the efficacy of the current treatment options. During infection, *C. difficile* produces toxins A and B, which are directly responsible for disease (6–9). As a result, the toxins have become promising nonantibiotic treatment targets. Despite the enormous significance of the toxins in *C. difficile* pathogenesis, the molecular mechanisms and key players involved in the regulation of the toxins are not fully understood. Knowledge of the mechanism of *C. difficile* toxin regulation will be critical in developing novel nonantibiotic therapies to target the toxins for the treatment of this pathogen of significant public health importance.

The accessory gene regulator (Agr) quorum signaling system regulates virulence and colonization factors in several Gram-positive bacteria (3, 10, 11). In *Staphylococcus aureus*, where it has been excellently characterized, the Agr system is transcribed as a four-gene operon comprising *agrA*, -*B*, -*C*, and -*D* (12–14). The *agrD* gene encodes a prepeptide that is processed by the transmembrane protein AgrB to produce an autoinducer peptide (AIP) and released into the extracellular milieu. The AgrC histidine kinase protein senses and binds extracellular AIP, which activates its ATPase activity, resulting in autophosphorylation and phosphoryl group transfer to AgrA. Phosphorylated AgrA forms a dimer, which enables the C-terminal DNA binding domain to bind either to the promoter region of the *agr* operon to produce more transcripts of the *agr* system components or at the RNAIII promoter to regulate the transcription of target genes (10, 15).

The *agr* operon can commonly be divided into two pathways: (i) a signal generation pathway that generates the AIP and (ii) a response pathway that senses and transduces the AIP to activate target gene expression. Some *C. difficile* strains possess two loci of the *agr* component genes, designated *agr1* and *agr2*. The *agr1* locus is present in all of the *C. difficile* strains sequenced to date (including the historical 630 strain) and contains only the AIP generation pathway genes (*agrB1* and *agrD1*). On the other hand, the *agr2* locus, which is mostly present in a small number of strains, including the hypervirulent NAP1/027 R20291 strain, contains both AIP generation (*agrB2*, *agrD2*) and AIP response (*agrC2*, *agrA2*) genes. The main roles of these two accessory gene loci in *C. difficile* pathogenesis have not been clear until now. We previously demonstrated that *C. difficile* toxins A and B are regulated by an Agr-like quorum signaling system and purified a peptide thiolactone AIP (termed the TI signal) from the stationary-phase culture supernatant that activates toxin production (16). Because of the presence of the *agr1* locus in the genomes of all sequenced toxigenic *C. difficile* strains, we hypothesized that it is responsible for TI signal generation and plays a central role in toxin production. Using allelic exchange, we deleted the *agr* loci from the 630 and NAP1/027 R20291 strains, and the results demonstrate that the *agr1* mutant of each strain is avirulent. This important finding underscores the clinical implication of Agr quorum signaling in *C. difficile* pathogenesis and opens up a unique therapeutic target for the development of a novel nonantibiotic therapy for CDI.

## RESULTS

**FIG 1:**
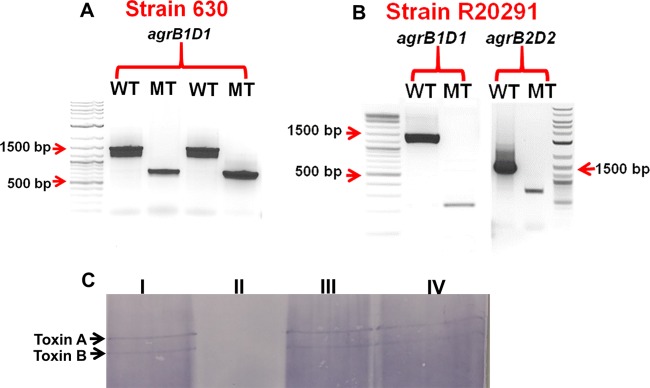
Deletion of the *C. difficile* accessory gene regulator autoinducing peptide generation loci (*agrB1D1* and *agrB2D2*) by allelic exchange. DNA sequences upstream and downstream of the *agrB1D1* locus of strain 630 (A) and the *agrB1D1* and *agrB2D2* loci of strain R20291 (B) were cloned into the pMTL82151 plasmid and transferred into the *C. difficile* strains by conjugation with *E. coli* CA434 bearing the assembled allelic-exchange cassette. The deletion mutants were confirmed by PCR and analyzed by 1% agarose gel electrophoresis. (C) Western blot analysis of 48-h culture supernatant fluids from the R20291 *agrB1D1* and *agrB2D2* mutants. Supernatants from 48-h cultures were concentrated with the Pierce 150-kDa concentrator (Thermo Fisher Scientific Inc., Rockford, IL) and subjected to 6% PAGE. The protein bands were transferred onto a 0.45-µm nitrocellulose membrane and probed with monoclonal antibodies specific for toxins A and B. The toxin bands on the transferred membrane were detected with the Protein Detector Western blot BCIP/NBT kit (KPL, Gaithersburg, MD). WT, wild type; MT, mutant; I, wild type; II, *agrB1D1* mutant; III, complemented *agrB1D1* mutant; IV, *agrB2D2* mutant.

To examine the roles of the two accessory gene regulator loci in *C. difficile* toxin production and virulence, the proposed AIP generation pathway loci *agrB1D1* and *agrB2D2* were deleted by allelic exchange. DNA sequences upstream and downstream of these loci (see Fig. S1 in the supplemental material) were PCR amplified, assembled, and cloned into plasmid pMTL82151 (17). Following conjugation, single-crossover mutants were selected by subculturing three times in a medium with antibiotic selection. Double-crossover mutants were subsequently selected by subculturing the single-crossover mutants 10 times without antibiotic selection and the deletion mutants were confirmed by PCR. Deletion of the *agrB1D1* locus resulted in a PCR product of 756 bp, compared to 1,500 bp for wild-type strain 630 and 258 bp (wild type, 1,500 bp) for strain R20291 (Fig. 1A and B). Deletion of the *agrB2D2* locus from strain R20291 also resulted in a 560-bp (wild type, 1,340 bp) PCR product. To confirm the locations of the deletions in the genome, the PCR products were sequenced and the DNA sequence results demonstrated complete deletion of the *agrB1D1* locus from the genomes of both strains and of the *agrB2D2* locus from that of strain R20291.

### *C. difficile* R20291 and 630 *agrB1D1* mutants cannot produce toxins.

To evaluate the effects of *agrB1D1* and *agrB2D2* deletions on toxin production, 48-h culture supernatant fluids collected from the mutant and wild-type strains were concentrated and analyzed by Western blotting with monoclonal antibodies that specifically recognize toxins A and B. No toxin bands were detected in the culture fluids collected from the R20291 *agrB1D1* mutant (Fig. 1C) and the 630 *agrB1D1* mutant (see Fig. S2A in the supplemental material). However, the bands corresponding to toxins A and B were detected in the culture supernatant fluids collected from the R20291 wild-type strain and the *agrB2D2* mutant. The culture fluids were further tested for the presence of toxins by enzyme-linked immunosorbent assay (ELISA), and the results corroborated those obtained by Western blotting (see Fig. S2B and C). To examine whether the type of growth medium may affect toxin production by the mutants, the cells were also cultured in TY medium (3% Bacto tryptose, 2% yeast extract) and the supernatant fluids were tested for toxin. No toxin was detected in the supernatant fluids collected from the *agrB1D1* mutants of both strains (see Fig. S3), suggesting that the function of the *agrB1D1* locus in toxin production is not affected by the type of growth medium.

**FIG 2:**
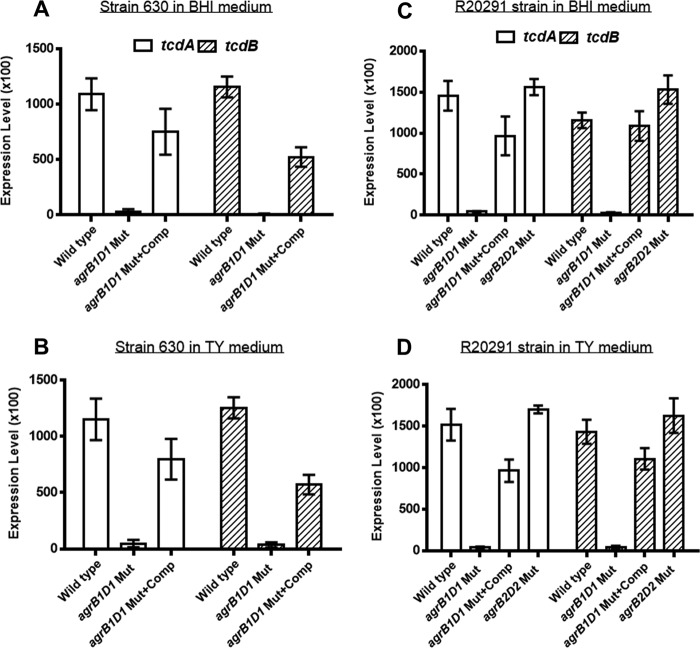
Analysis of *agr* mutants for transcription of the toxin genes (*tcdA* and *tcdB*) in BHI and TY media. The 630 wild-type, *agrB1D1* mutant, and complemented *agrB1D1* mutant strains were cultured in either BHI (A) or TY (B) medium. The R20291 wild-type, *agrB1D1* mutant, complemented *agrB1D1* mutant, and *agrB2D2* mutant strains were also cultured in either BHI (C) or TY (D) medium. The cultures were incubated for 16 h anaerobically at 37°C. Total RNA was isolated with the RNeasy kit (Qiagen), and this was followed by cDNA synthesis by reverse transcription using the ProtoScript AMV First Strand cDNA synthesis kit (New England Biolabs, Ipswich, MA) with 1 µg of the isolated total RNA. Quantitative PCR was performed with primers specific for *tcdA*, *tcdB*, and the cDNA used as the template. Known quantities of *tcdA* and *tcdB* DNA were used as standards. The difference between the *tcdA* and *tcdB* transcript levels detected in the wild-type and *agrB1B1* mutant strains was significant (*P* = 0.0001), but there was no significant difference (*P* = 0.235) between the two culture media. Error bars represent the standard deviations of three independent experiments.

To examine whether the *agr* mutants transcribe the toxin genes, total RNA was isolated from mid-stationary-phase cultures of the mutants grown in either brain heart infusion (BHI) medium or TY medium and quantitative PCR was performed with cDNA converted from total RNA as the template. No significant mRNA transcripts corresponding to the toxin genes (*tcdA* and *tcdB*) were detected from the 630 and R20291 *agrB1D1* mutants cultured in either BHI or TY medium (Fig. 2A to D). On the contrary, the toxin gene transcripts were detected in the wild-type strains and R20291 *agrB2D2* mutant cultured in both media. Furthermore, there was no significant difference (*P* = 0.235) in the mRNA transcripts detected in the mutants cultured in both media. These results demonstrate that the *agrB1D1* mutant is unable to transcribe significant amount of the toxin genes and that the *agrB1D1* locus plays a central role in toxin production.

**FIG 3:**
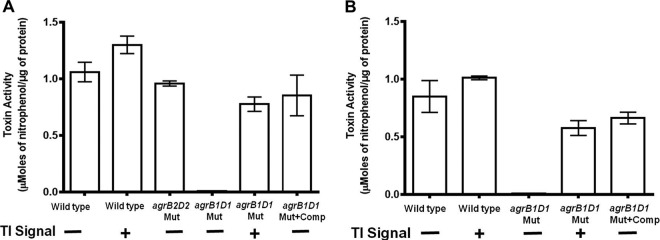
Plasmid-mediated and extracellular complementation restores toxin production in the hypervirulent NAP1/027 R20291 (A) and nonhypervirulent 630 (B) toxin-deficient *agrB1D1* mutants. The R20291 and 630 *agrB1D1* mutants were complemented with a plasmid bearing the wild-type *agrB1D1* locus, and the complemented mutants were tested for toxin production. The mutants were also incubated in BHI medium anaerobically for 48 h in the presence or absence of the TI signal purified from wild-type strain 630. Toxin production was detected with the Cdifftox activity assay (30). *agrB2D2* Mut, R20291 *agrB2D2* deletion mutant; *agrB1D1* Mut, *agrB1D1* deletion mutant; *agrB1D1* Mut+Comp, *agrB1D1* deletion mutant complemented with a plasmid bearing the wild-type *agrB1D1* locus. The difference in toxin activity between the wild type and the *agrB1D1* mutant was significant (*P* = 0.0001 for both strains). Error bars represent the standard deviations of three independent experiments.

To confirm that loss of toxin production by the mutants was directly due to the absence of the *agrB1D1* genes, the mutants were transformed with a plasmid bearing the wild-type *agrB1D1* locus. This restored toxin production in both 630 and R20291 *agrB1D1* mutants at levels comparable to those of the wild-type strains (Fig. 3A and B), indicating that *agrB1D1*, which is universally present in all of the *C. difficile* strains whose genomes have been sequenced to date, is essential in toxin production.

We previously purified a thiolactone AIP (designated the TI signal) from strain 630 and R20291 stationary-phase culture supernatant fluids that induced toxin production in all strains (16). Bioinformatic analysis suggested that the TI signal originates from the *agrD1* gene, which is a component of the *agrB1D1* locus. To investigate whether the purified TI signal can restore toxin production in R20291 and 630 *agrB1D1* mutants, these mutants were incubated with the TI signal and their culture fluids were tested for toxin. In the absence of the TI signal, no toxin was detected in the culture fluids collected from both R20291 and 630 *agrB1D1* mutants (Fig. 3A and B). Remarkably, toxin was detected when the *agrB1D1* mutants were cultured in the presence of the TI signal, indicating that the TI signal restored the ability of the *agrB1D1* mutants to make toxins.

To determine whether *agrB1D1* mutants can produce the TI signal, culture supernatant fluids from the mutants were examined for the presence of the TI signal by a previously described high-performance liquid chromatography (HPLC)-based purification method (16). No HPLC peak associated with the TI signal was observed in the culture fluids from 630 and R20291 *agrB1D1* mutants. Likewise, fractions collected at elution times similar to that of the TI signal did not induce toxin production. On the other hand, the TI signal peak was observed in the culture fluids collected from either the complemented *agrB1D1* mutants or the R20291 *agrB2D2* mutant that has a wild-type *agrB1D1* locus. HPLC fractions associated with the observed TI signal peak induced toxin production in both 630 and R20291 *agrB1D1* mutants (see Fig. S4A and B in the supplemental material). These series of experiments provide direct evidence that the *agrB1D1* mutant is deficient in TI signal synthesis and that the *agrB1D1* locus is responsible for TI signal generation.

### Culture supernatant fluids from 630 and R20291 *agrB1D1* mutants do not cause cytotoxicity and have no effect on cell viability.

**FIG 4:**
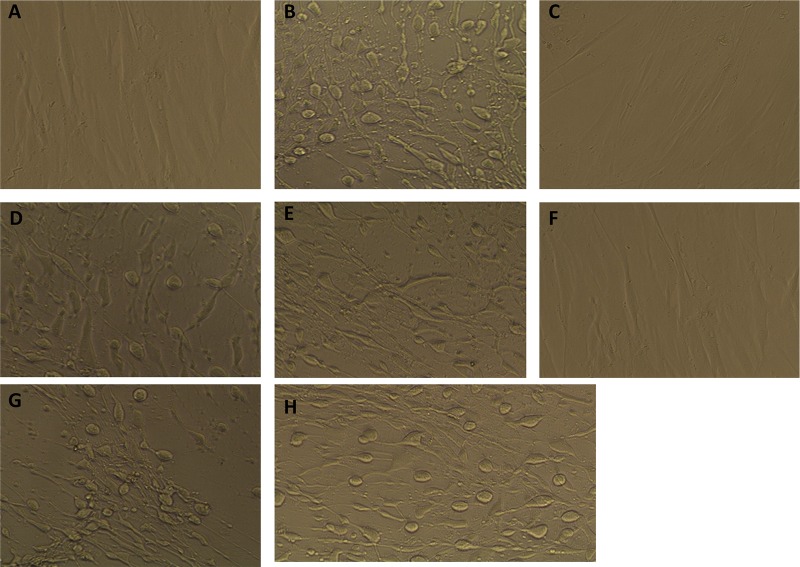
Culture supernatant fluids collected from the *agrB1D1* mutants are not cytotoxic to human foreskin fibroblast cells. Strain 630 and R20291 *agrB1D1* and *agrB2D2* mutants were cultured for 48 h in BHI medium anaerobically at 37°C. The culture supernatant fluids were collected, filter sterilized with a 0.45-µm filter, and examined for cytotoxicity with the Bartels *Clostridium difficile* cytotoxicity assay kit (Trinity Biotech, Jamestown, NY). The culture fluids were incubated with the fibroblast cells for 24 h and observed under a microscope for cytotoxic effects. Images were taken with an EVOS XL microscope (Advanced Microscopy Group) at ×20 magnification. Panels: A, a representative image of fibroblast cells cultured in growth medium only; B, wild-type 630; C, 630 *agrB1D1* mutant; D, 630 complemented *agrB1D1* mutant; E, wild-type R20291; F, R20291 *agrB1D1* mutant; G, complemented R20291 *agrB1D1* mutant; H, R20291 *agrB2D2* mutant.

The *C. difficile* toxins are cytotoxic and cause cell rounding when incubated with human foreskin fibroblast cells. To investigate whether culture supernatant fluids from the mutants can cause cytotoxicity, human foreskin fibroblast cells were incubated with sterilized 48-h culture fluids collected from the *agr* mutant strains and analyzed by microscopy for pathologic effects. As expected, fibroblast cells incubated with culture fluids collected from the wild-type 630 and R20291 strains and the R20291 *agrB2D2* mutant appeared rounded and spindle-like and generally exhibited altered morphology (Fig. 4B and E and H); all of these phenotypes are consistent with previous reports describing the cytopathic and cytotoxic effects of the toxins (11, 18, 19). On the contrary, fibroblast cells incubated with culture fluids from the 630 and R20291 *agrB1D1* mutants did not exhibit either cytopathic or cytotoxic effects and appeared morphologically similar to untreated control fibroblast cells (Fig. 4A, C, and F). The cytotoxic effects associated with the toxins were observed when fibroblast cells were treated with culture fluids from the complemented 630 and R20291 *agrB1D1* mutants that harbored a plasmid-borne wild-type *agrB1D1* locus (Fig. 4D and G).

**FIG 5:**
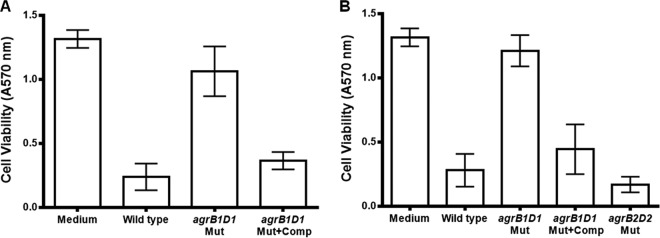
Culture supernatants from *C. difficile agrB1D1* mutants have no effect on cell viability. Strain 630 (A) and R20291 (B) *agrB1D1* and *agrB2D2* mutants were cultured for 48 h in BHI medium anaerobically at 37°C. The culture supernatant fluids were collected, filter sterilized with a 0.45-µm filter, and tested for their effect on cell viability. Briefly, ready-made fibroblast cells (Trinity Biotech, Jamestown, NY) were treated with the culture supernatant fluids and incubated for 48 h at 37°C aerobically. Following the incubation period, cell viability was examined with the CellTiter 96 nonradioactive cell proliferation assay (Promega, Madison, WI) in accordance with the instructions provided by the manufacturer. *agrB1D1* Mut, *agrB1D1* deletion mutant; *agrB1D1* Mut+Comp, *agrB1D1* mutant complemented with a plasmid bearing the wild-type *agrB1D1* locus; *agrB2D2* Mut, R20291 *agrB2D2* deletion mutant. The differences between the amounts of formazan produced by fibroblast cells treated with supernatants from the *agrB1D1* mutants and wild-type strains, the complemented *agrB1D1* mutants, and the R20291 *agrB2D2* mutant were significant (*P* = 0.0012). Error bars represent the standard deviations of three independent experiments.

To assess the viability of the treated fibroblast cells, the inherent ability of living cells to convert tetrazolium salt into a blue formazan product was measured using the CellTiter 96 Non-Radioactive Cell Proliferation Assay (Promega, Madison, WI). In this assay, the amount of tetrazolium salt conversion to formazan corresponds to the number of viable cells. The results showed no significant difference (*P* = 0.305) between the amount of formazan produced by fibroblast cells incubated with culture fluids from the 630 and R20291 *agrB1D1* mutants and that produced by untreated control cells (Fig. 5A and B). Similarly, the amount of formazan produced by fibroblast cells treated with culture supernatants from the complemented 630 and R20291 *agrB1D1* mutants, the R20291 *agrB2D2* mutant, and the respective wild-type strains were also comparable and not significantly different (*P* = 0.356). However, the amount of formazan produced by fibroblast cells treated with culture fluids collected from the *agrB1D1* mutants and untreated controls was significantly higher (*P* = 0.001) than that produced by the wild-type, complemented *agrB1D1* mutant, and R20291 *agrB2D2* mutant strains. These results demonstrate that the culture fluid from the *agrB1D1* mutant is not cytotoxic and this is likely due to the absence of toxin.

### R20291 *agrB1D1* mutant is avirulent in mice.

**FIG 6:**
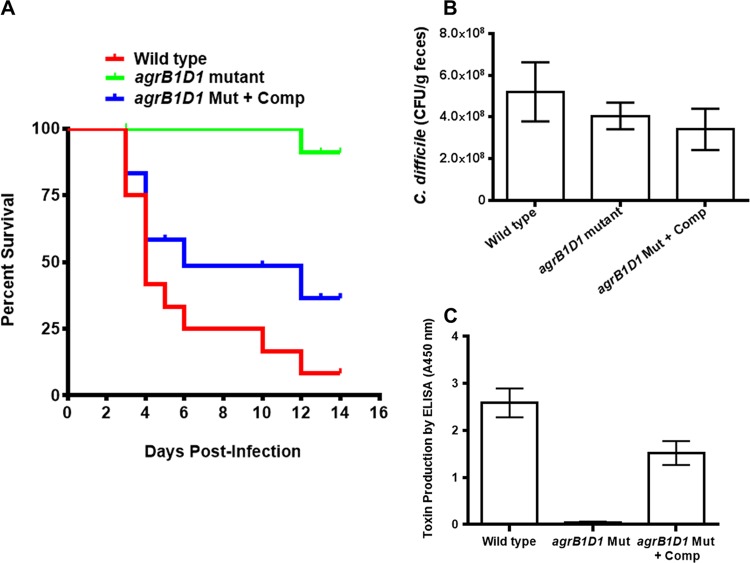
Hypervirulent R20291 *agrB1D1* mutant is unable to cause *C. difficile*-associated disease in mice. Seven-week-old (both male and female) C57BL/6 mice (*n* = 12 in each group) were first treated with a cocktail of antibiotics; infected with the R20291 wild type, *agrB1D1* mutant, *agrB2D2* mutant, or complemented *agrB1D1* mutant; and then monitored for 14 days. The animals were scored on the basis of the following endpoint symptoms: (i) diarrhea, hunched posture, and physical appearance (0, normal [good hair coat, normal posture, frequent grooming, no discharge from the eyes or nose]; 1, hunched posture and infrequent grooming; 2, hunched posture, no grooming [ruffled hair], distended stomach, and diarrhea [noticeable dehydration]); (ii) movement and response to external stimuli (0, normal [normal activity, alertness, response to stimuli, regular eating and drinking habits]; 1, reduced movement, reduced alertness, reduced response to stimuli, or uncoordinated movement; 2, no movement, no activity, no eating, no drinking or alertness); and (iii) body weight changes (0, normal [normal food consumption with normal urine and fecal output]; 1, 5 to 15% loss of body weight; 2, 15 to 20% loss of body weight). Animals that were assessed an endpoint score of 2 for any of the above three parameters during the 14-day observation period were euthanized because they were always moribund. Percent survival is the proportion of animals assessed an endpoint score of <2. (B) colonization of mice by the *agrB1D1* mutant and the complemented mutant. (C) No toxins were detected in pellets of mice infected with the R20291 *agrB1D1* mutant. Pellets collected from the mice on day 5 were tested for the presence of *C. difficile* cells by counting the CFU on selective plates and for toxins A and B with the Wampole *C. difficile* TOX A/B II assay (Technologies Lab, Blacksburg, VA). Data presented are the average readings from the 12 mice evaluated per group. The difference between the survival rates of mice treated with the *agrB1D1* mutant and the wild type was statistically significant (*P* = 0.0001). There was no significant difference (*P* = 0.439) between the CFU counts of mice infected with the wild-type strain, the *agrB1D1* mutant, or the complemented *agrB1D1* mutant. Error bars represent the standard deviations of the 12 samples tested per group.

The hypervirulent *C. difficile* NAP1/027 R20291 strain is known to cause severe disease due to high toxin production, and it is commonly isolated from stool samples of CDI patients (20–23). To evaluate whether the *agr* mutant can cause disease in an oral CDI infection model, mice were infected with wild-type R20291, the *agrB1D1* mutant, and the complemented *agrB1D1* mutant. The animals were monitored for 14 days postinfection and scored on the basis of three endpoint symptoms: (i) diarrhea, hunched posture, and physical appearance; (ii) movement and response to external stimulus; and (iii) weight loss. Mice (*n* = 12 in each group) infected with the wild-type strain became moribund from day 2, and only 8% survived for 14 days (Fig. 6A). Of the mice infected with the R20291 *agrB1D1* mutant, 92% survived the 14-day postinfection monitoring period. The difference between the survival rates of mice treated with the *agrB1D1* mutant and mice treated with the wild type was statistically significant (*P* = 0.0001). However, when the mice were infected with the complemented *agrB1D1* mutant, 42% survived and the survival rate was not statistically significantly different from that of mice treated with the wild-type strain (*P* = 0.2206).

To examine the ability of the *agrB1D1* mutant to colonize, collected stool pellets were diluted and cultured on selective plates for CFU counting. There was no significant difference (*P* = 0.335) between the CFU counts of mice infected with the wild-type strain, mice infected with the *agrB1D1* mutant, and mice infected with the complemented *agrB1D1* mutant (Fig. 6B). These results demonstrate that the *agrB1D1* mutant is able to colonize, similar to the wild type. The collected stool pellets were also tested for toxin by ELISA. No toxin was detected in the pellets from the animals infected with the *agrB1D1* mutant. On the other hand, toxins were detected in all of the mice infected with the wild-type and complemented *agrB1D1* mutant strains (Fig. 6C). All together, these results provide further evidence that the *agrB1D1* locus is essential for *C. difficile* virulence and underscore the importance of the Agr quorum signaling system in the pathogenesis of this multidrug-resistant pathogen.

## DISCUSSION

Recent years have seen an increase in the incidence of CDIs related to antibiotic use (3). Broad-spectrum antibiotic therapy is a major risk factor for CDI (2). With increasing resistance to antibiotic treatment of CDIs, patients are experiencing higher costs of health care and a lower quality of life as treatment options decrease (21, 24–26). These problems, along with reduced colonization resistance associated with alteration of the gut microbiota, make long-term treatment of recurrent CDIs with antibiotics ineffective. As a result, there is a new interest in developing effective nonantibiotic therapies for CDIs. Since the A and B toxins are directly associated with disease, targeting these toxins by inhibiting either their production or their activity is deemed a promising approach. Antimicrobial resistance is expected to be greatly reduced because of the lack of selective pressure with this approach. In fact, several studies have proposed using nontoxigenic *C. difficile* strains to outcompete toxigenic strains as a treatment method (27). Here, we have identified a bona fide *C. difficile* toxin regulatory pathway that can be targeted therapeutically as a nonantibiotic therapy for CDI.

Using a genetic approach, we systematically deleted the *agrB1D1* and *agrB2D2* quorum signaling loci from the *C. difficile* 630 and R20291 strains and evaluated the mutants for toxin production. Our results demonstrate that the *agrB1D1* locus is responsible for generating the TI signal, a peptide thiolactone required to activate toxin production (16). We further show that the *agrB1D1* mutant is unable to produce toxin because it cannot generate the TI signal. The *agrB1D1* locus is present in all of the toxigenic *C. difficile* strains whose genomes have been sequenced to date, unlike the *agrB2D2* locus that is present in a few strains. In the NAP1/027 R20291 strain that has both the *agrB1D1* and *agrB2D2* loci in its genome (see Fig. S1 in the supplemental material), only *agrB1D1* deletion leads to loss of toxin production, suggesting that the *agrB1D1* locus plays a central role in *C. difficile* toxin A and B production. An *agrB2D2* deletion mutant that has the wild-type *agrB1D1* genes is able to generate the TI signal and thus produce toxin (Fig. 3). Moreover, the purified TI signal from the R20291 *agrB2D2* mutant is able to activate toxin production in the 630 and R20291 *agrB1D1* mutants (see Fig. S3). We have also demonstrated that the R20291 *agrB1D1* mutant is able to colonize, but unable to cause disease in a mouse model of CDI. These findings provide strong support for the roles of the *agrB1D1* locus and toxin production in *C. difficile* pathogenesis and virulence.

We note that our attempt to generate an isogenic mutant *agrD1* gene was unsuccessful because of its small size (147 bp). However, the AgrB1 protein is required to process and cleave the AgrD1 autoinducing prepeptide to generate the TI signal, and hence, deletion of the *agrB1D1* locus was the most plausible alternative. Also, Martin et al. (28) observed a modest decrease in toxin A level in the R20291 strain with a TargeTron insertion in *agrA2*. The basis for the slightly different results is unclear at present. Also, the *agr1* locus has no cognate two-component sensor histidine kinase and response regulator in its vicinity in the genome, as would be expected in other quorum signaling systems. We previously demonstrated that the R20291 strain utilizes the two-component response regulator *agrA2* present at the *agr2* locus to transduce the TI signal (16), now established to be produced solely from its *agr1* locus. However, how other strains, such as 630, that do not have the *agr2* locus in their genomes sense and transduce the TI signal has yet to be determined. Our investigations to identify all of the key players of the two *C. difficile* Agr quorum signaling systems and their respective roles are ongoing. We anticipate that identification and characterization of the TI signal sensory and response regulatory elements in all of the strains will likely provide evolutionary insights into this unique toxin regulatory system. Overall, our current findings have uncovered a promising target for the development of a novel nonantibiotic therapy to combat *C. difficile*, a multidrug-resistant pathogen of significant public health importance.

## MATERIALS AND METHODS

### Bacterial strains and growth conditions.

Toxigenic *C. difficile* strain ATCC BAA-1382 (*tcdA*^+^ *tcdB*^+^; strain 630) was purchased from the American Type Culture Collection (Manassas, VA), and the R20291 stock (NAP1/027; *tcdA*^+^ *tcdB*^+^) was from a previous study (16). Bacterial cultures were grown in BBL BHI medium (Becton, Dickinson, Cockeysville, MD) or TY medium. Single colonies were also isolated on Cdifftox agar (29) plates. Cultures were incubated anaerobically in an atmosphere of 10% H_2_, 5% CO_2_, and 85% N_2_ at 37°C in a Controlled Atmosphere Anaerobic Chamber (PLAS LABS, Lansing, MI). The BHI medium used in all of the experiments was reduced by incubation overnight in the anaerobic chamber prior to use. The substrate for the Cdifftox activity assay (30), *p*-nitrophenyl-β-d-glucopyranoside, was purchased from Biosynth International (Itasca, IL).

### Sample storage conditions for bacterial stocks.

Bacterial stocks were stored short-term in chopped meat broth (BD Diagnostics, Franklin Lakes, NJ) at room temperature or long-term in autoclaved 10% dimethyl sulfoxide at −80°C.

### Toxin assays. (i) Cdiff activity assay.

The Cdifftox activity assay (30) was used to detect the combined activities of *C. difficile* toxins A and B in culture supernatant fluid. Briefly, the culture was centrifuged for 15 min at 10,000 × *g* at 4°C; 250 µl of the supernatant fluids was incubated with 30 µl of 30 mM *p*-nitrophenyl-β-d-glucopyranoside and incubated either for 4 h at 37°C or overnight at room temperature. The assay was quantitated spectrophotometrically by reading the absorbance of 410 nm. Total protein concentrations were determined with the Pierce BCA protein assay kit (Thermo Fisher Scientific Inc.). A molar extinction coefficient (ε) of 17,700 M^−1^ cm^−1^ for *p*-nitrophenol (31) was used for the calculation of the number of micromoles of *p*-nitrophenol produced per microgram of protein.

### (ii) ELISA.

The presence of toxins A and B in the culture supernatants was also tested with the Wampole *C. difficile* TOX A/B II assay (Technologies Lab, Blacksburg, VA). This assay was performed by the protocol provided by the manufacturer.

### Cytotoxicity assay.

The effect of the culture supernatant on cytotoxicity was tested with the Bartels *Clostridium difficile* cytotoxicity assay kit (Trinity Biotech, Jamestown, NY). The protocol used was based on the instructions provided by the manufacturer. Briefly, human foreskin fibroblast cells were incubated for 24 to 48 h at 37°C with 48-h culture supernatant fluids collected from the wild-type 630 and R20291 strains and the respective *agrB1D1* and *agrB2D2* mutants. Cells were observed with an EVOS XL microscope (Advanced Microscopy Group, Bothell, WA).

### Cell viability assay.

The culture supernatants from the wild-type 630 and R20291 strains and the respective *agr* mutants were tested for their effects on foreskin fibroblast cell viability with the CellTiter 96 Non-Radioactive Cell Proliferation Assay (Promega, Madison, WI). This assay measures the ability of living cells to convert tetrazolium salt into a blue formazan product, which can be measured with a spectrophotometer. Ready-made human foreskin fibroblast cells (Trinity Biotech, Jamestown, NY) in 150 µl of medium were treated with 50 µl of culture supernatant fluids from the wild-type and *agr* mutant strains and incubated for 48 h at 37°C. Following the incubation period, 100 µl of the supernatant was removed and replaced with 15 µl of the dye solution and the cells were incubated for 4 h at 37°C. Solubilization Stop Mix Solution (100 µl) was then added and mixed thoroughly to obtain a uniformly colored solution. Absorbance at 570 nm was measured. A reference wavelength of 650 nm was used to reduce background noise contributed by cell debris and other nonspecific absorbance.

### Allelic-exchange-based deletion of the *agrB1D1* and *agrB2D2* loci.

The allele exchange cassette used for deletion of the *agrB1D1* and *agrB2D2* loci from the 630 and R20291 strains were constructed in pUC57. To create the cassette for the *agrB1D1* locus, 1,527- and 923-bp sequences homologous to the DNA sequences flanking the upstream and downstream regions of the locus, respectively, were amplified with primers RH-AGRB1D1-F, RH-AGRB1D1-R, LH-AGRB1D1-F, and LH-AGRB1D1-R (see Table S1 in the supplemental material). SacI and KasI recognition sequences were incorporated into the primers at the 5′ and 3′ ends of the 1,527 bp upstream of the locus. The 923-bp sequences downstream of the locus also contained KasI and BamHI recognition sequences at the 5′ and 3′ ends. PCR amplification was performed with OneTaq Quickload PCR Mastermix (NEB, Ipswich, MA) and genomic DNA from the 630 or R20291 strain. The PCR fragments were assembled in plasmid pUC57 by using the Sac1 and BamHI sites. The assembled left and right flanking regions of the *agrB1D1* locus were inserted into replication-defective *C. difficile* vector pMTL82151 (kindly provided by Alexandra Faulds-Pain) by using the Sac1 and BamHI sites, resulting in allelic-exchange cassette pAgrB1D1. For the *agrB2D2* cassette, 1,118- and 939-bp regions upstream and downstream of the *agrB2D2* locus were amplified with primers LH-AgrA2-F, LH-AgrA2-R, RH-AgrB2D2-F, and RH-AgrB2D2-R containing Sac1, Kas1, and BamHI restriction sites. The amplified fragments were assembled in the pBSK(+) Simple-Amp vector and subsequently cloned into pMTL82151, resulting in allelic-exchange cassette pAgrB2D2. Both the pAgrB1D1 and pAgrB2D2 assembled allelic-exchange cassettes were sequenced to confirm the absence of unintended mutations.

To generate the deletion mutants, *C. difficile* R20291 and 630 cells were incubated with conjugative *Escherichia coli* CA434 cells bearing either the pAgrB1D1 or the pAgrB2D2 allelic-exchange vector. The conjugants were plated on Cdifftox agar plates (29) without 5-bromo-4-chloro-3-indolyl-β-d-galactopyranoside but containing 250 µg/ml d-cycloserine, 8 µg/ml cefoxitin, and 50 µg/ml chloramphenicol. Conjugative *E. coli* CA434 cannot grow in the presence of d-cycloserine and cefoxitin, and so they were eliminated. Transconjugants were pooled and subcultured three times on the selective medium to obtain single-crossover clones. To allow more opportunity for the second recombination event to occur and to obtain double-crossover clones, the single-crossover clones were serially subcultured without chloramphenicol selection for 10 consecutive days. Double-crossover recombinants were identified by replica plating colonies onto nonselective BHI and BHI medium with chloramphenicol to screen for the loss of the plasmid-specific antibiotic resistance marker. Deletion of the *agrB1D1* and *agrB2D2* loci from the double-crossover clones was confirmed by PCR and sequencing 200 bp upstream and downstream of the fusion joint. The confirmation primers used were B1D1DelCon1-F and B1D1DelCon1-R for *agrB1D1* locus deletion and B2D2-CON-F and B2D2-CON-R for the *agrB2D2* locus. The PCR confirmation was performed with OneTaq Quick-Load 2× master mix (New England Biolabs) under the following conditions: initial denaturation at 94°C for 30 s and 36 cycles of 94°C for 30 s, 55°C for 30 s, and 68°C for 120 s. The PCR products were separated by 1% agarose gel electrophoresis and stained with ethidium bromide.

For complementation studies, the entire regions of the *agrB1D1* and *agrB2D2* loci were PCR amplified from wild-type R20291 and 630 DNA templates with oligonucleotides containing EcoRI and XbaI restriction sites (see Table S1 in the supplemental material). The digested PCR product was inserted into pMTL84151 (kindly provided by Alexandra Faulds-Pain). The plasmid was confirmed by DNA sequencing and conjugated into *C. difficile* R20291 and 630 *agrB1D1* mutants as described above. DNA sequencing was performed at Lone Star Labs (Houston, TX).

### Immunoblot analysis.

Culture supernatant fluids from 48-h cultures of the *agr* mutants and the wild type were concentrated with the Pierce 150-kDa Concentrator (Thermo Fisher Scientific Inc., Rockford, IL), and total protein concentrations were determined with the Pierce BCA protein assay kit (Thermo Fisher Scientific Inc.). The concentrated supernatant fluids (100-µg protein each) were separated by 6% polyacrylamide gel electrophoresis (PAGE) and transferred onto a 0.45-µm nitrocellulose membrane (Fisher Scientific Inc.) with a Trans-Blot cell (Bio-Rad) transfer apparatus. The membrane was blocked overnight in 10 mM Tris-buffered saline with 0.05% Tween 20 (TBST) containing 5% skim milk. Following blocking, the membrane was incubated with mouse monoclonal antibodies specific for *C. difficile* toxins A and B (Abcam, Cambridge, MA). Blots were washed three times (5 min each) in TBST between incubations. The Protein Detector Western blot BCIP/NBT kit (KPL, Gaithersburg, MD) was then used to probe the membrane for the presence of each toxin with an alkaline phosphatase-conjugated goat anti-mouse IgG secondary antibody, followed by incubation with the BCIP/NBT substrate, according to the manufacturer’s instructions.

### Analysis of mutants for transcription of *tcdA* and *tcdB* by quantitative reverse transcription-PCR.

The 630 and R20291 *agr* mutants at an optical density at 600 nm of 0.6 were diluted 1:100 in 30 ml of reduced BHI or TY medium and incubated anaerobically at 37°C for 16 h. Total RNA was isolated with the RNeasy kit (Qiagen) according to the manufacturer’s directions. The total RNA (1 µg of each) was converted to cDNA by reverse transcription with the ProtoScript AMV First Strand cDNA synthesis kit (New England Biolabs, Ipswich, MA) according to the manufacturer’s instructions. Relative expression levels of *tcdA* and *tcdB* transcripts were determined with SYBR green JumpStart Taq Ready Mix (Sigma), gene-specific primers (see Table S1 in the supplemental material), and 2 µl of cDNA. The SYBR green reaction mixtures contained each primer at 500 nM in a final volume of 20 µl. Known *tcdA* and *tcdB* DNA samples were used as standards. Comparative threshold cycle analysis was performed, and the mean expression levels obtained with three biological replicates. The absolute quantitative method was used to calculate the levels of *tcdA* and *tcdB* transcripts. Controls included the *rpoB* gene as an internal control and samples of the RNA preparation processed without the reverse transcription step, which uniformly yielded no detectable SYBR green signal.

### The murine CDI model.

Seven-week-old C57BL/6 mice (both males and females) were given a cocktail of antibiotics (32) for 5 days in their drinking water. The antibiotics were kanamycin (40 mg/kg), gentamicin (3.5 mg/kg), colistin (4.2 mg/kg), metronidazole (21.5 mg/kg), and vancomycin (4.5 mg/kg). One day after the antibiotic treatment, the mice were given 1 mg of clindamycin by oral gavage. About 24 h following clindamycin treatment, the mice were infected with 10^8^ viable spores of the *agrB1D1* mutant, the complemented mutant, or the wild-type strain in a suspension of 250 µl of phosphate-buffered saline (PBS) by oral gavage. The animals were observed twice daily for 14 days postinfection. An endpoint scoring system was devised and used to evaluate the animals. A scoring chart based on three main parameters ([i] diarrhea, hunched posture, and physical appearance; [i] movement and response to external stimulus; and [iii] weight loss [described below]), was used to determine the endpoint. Diarrhea, hunched posture, and physical appearance were scored as follows: 0, normal (good hair coat, normal posture, frequent grooming, no discharge from the eyes or nose); 1, hunched posture and infrequent grooming; 2, hunched posture, no grooming (ruffled hair), distended stomach, and diarrhea (noticeable dehydration). Movement and response to external stimuli were scored as follows: 0, normal (normal activity, alertness, response to stimuli, usual eating and drinking); 1, reduced movement, reduced alertness, reduced response to stimuli, or uncoordinated movement; 2, no movement, no activity, no eating, no drinking or alertness. Body weight changes were scored as follows: 0, normal (normal food consumption with normal urine and fecal output); 1, 5 to 15% loss of body weight; 2, 15 to 20% loss of body weight. Animals that were assessed an endpoint score of 2 for any of the above three parameters during the 14-day observation period were euthanized.

Pellets collected from the mice on day 5 postinfection were tested for the presence of toxins A and B with the Wampole *C. difficile* TOX A/B II assay (Technologies Lab, Blacksburg, VA). Briefly, one to three stool pellets or watery diarrheal stool samples were collected from the anal region of each mouse, weighed, suspended in 250 µl of sterile PBS, and centrifuged at 10,000 × *g*. ELISA of the supernatant was performed, whereas the pellet was evaluated for the number of CFU by resuspension of the pellet in BHI, dilution, and plating on Cdifftox agar plates.

### Data analysis.

All of the data were analyzed and plotted with GraphPad Prism version 6.07 for Windows (GraphPad Software, San Diego, CA). The Student *t* test was used to compare differences between samples. A Kaplan-Meier curve was used to analyze the survival of the animals. In all cases, statistical significance was defined as a *P* value of <0.05.

## SUPPLEMENTAL MATERIAL

Figure S1  The *C. difficile* accessory gene regulator loci showing the location of the region deleted by allelic exchange. The *agr1* locus in strains 630 (A) and R20291 (B) and the *agr2* locus in strain R20291 (C) are shown. Download Figure S1, TIF file, 0.4 MB

Figure S2  (A) Western blot analysis of 48-h culture supernatant fluid from the 630 *agrB1D1* mutant. Supernatant from a 48-h culture was concentrated with the Pierce 150-kDa concentrator (Thermo Fisher Scientific Inc., Rockford, IL) and subjected to 6% PAGE. The protein bands were transferred onto a 0.45-µm nitrocellulose membrane and probed with monoclonal antibodies specific for toxins A and B. The toxin bands on the transferred membrane were detected with the Protein Detector Western blot BCIP/NBT kit (KPL, Gaithersburg, MD). I, wild type; II, *agrB1D1* mutant. (B) The *agr1* deletion abolishes toxin production in both the 630 (B) and R20291 (C) strains. The *agrB1D1* mutants of both strains and a *agrB2D2* mutant of the R20291 strain were incubated in BHI medium for 48 h anaerobically at 37°C. Toxin production was tested by ELISA with the Wampole *C. difficile* TOX A/B II assay (Technologies Lab, Blacksburg, VA). *agrB1D1* Mut, *agrB1D1* deletion mutant; *agrB1D1* Mut+Comp, *agrB1D1* mutant complemented with a plasmid bearing the wild-type *agrB1D1* locus; *agrB2D2* Mut, *agrB2D2* deletion mutant. There were significant differences (*P* = 0.0023 for 630 and 0.0001 for R20291) between the amounts of toxins produced by the wild-type and *agrB1D1* mutant strains. Error bars represent the standard deviations of three independent experiments. Download Figure S2, TIF file, 0.5 MB

Figure S3  The *agr1* mutants do not produce toxin in TY medium. *agrB1D1* mutants of both strains and the R20291 *agrB2D2* mutant strain were incubated in TY medium for 48 h anaerobically at 37°C. Toxin production was detected with the Cdifftox activity assay. *agrB2D2* Mut, R20291 *agrB2D2* mutant; *agrB1D1* Mut, *agrB1D1* mutant. There was a significant difference (*P* = 0.003 for 630 and 0.0001 for R20291) between the levels of toxin activity produced by the wild-type and *agrB1D1* mutant strains. Error bars represent the standard deviations of three independent experiments. Download Figure S3, TIF file, 0.3 MB

Figure S4  The R20291 *agrB2D2* mutant produces an active TI signal that restores toxin production in 630 and R20291 *agrB1D1* mutants unable to make toxins. 630 and R20291 *agrB1D1* mutant strains were incubated in BHI medium anaerobically for 24 h in the presence of the TI signal purified from the R20291 *agrB2D2* mutant. Toxin activity was detected with the Cdifftox activity assay (A), and toxin production was tested by nonquantitative ELISA (B) with the Wampole *C. difficile* TOX A/B II assay (Technologies Lab, Blacksburg, VA). *agrB1D1* Mut, *agrB1D1* deletion mutant; TI, TI signal. There were significant differences (*P* = 0.0041 for 630 and 0.0001 for R20291) between the amounts of toxins produced by the wild-type and *agrB1D1* mutant strains in the absence of the TI signal. Error bars represent the standard deviations of three independent experiments. Download Figure S4, TIF file, 0.5 MB

Table S1  Primers used in this study. Table S1, TIF file, 0.4 MB
