# Erratum for Darkoh et al., “Accessory Gene Regulator-1 Locus Is Essential for Virulence and Pathogenesis of *Clostridium difficile*”

**DOI:** 10.1128/mBio.01643-17

**Published:** 2017-10-31

**Authors:** Charles Darkoh, Chioma Odo, Herbert L. DuPont

**Affiliations:** a Department of Epidemiology, Human Genetics, and Environmental Sciences, Center For Infectious Diseases, University of Texas Health Science Center, School of Public Health, Houston, Texas, USA; b Microbiology and Molecular Genetics Program, University of Texas Graduate School of Biomedical Sciences, Houston, Texas, USA

## ERRATUM

Volume 7, no. 4, e01237-16, 2016, https://doi.org/10.1128/mBio.01237-16. In Fig. 4, panel A was a duplicate of panel F. The figure has been revised. The correct image for panel A is shown below.

**FIG 4:**
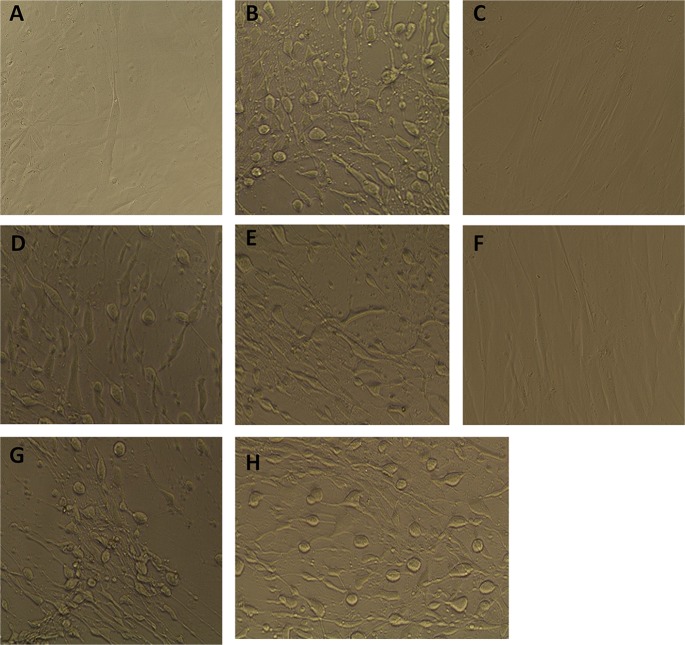
Culture supernatant fluids collected from the *agrB1D1* mutants are not cytotoxic to human foreskin fibroblast cells. Strain 630 and R20291 *agrB1D1* and *agrB2D2* mutants were cultured for 48 h in BHI medium anaerobically at 37°C. The culture supernatant fluids were collected, filter sterilized with a 0.45-µm filter, and examined for cytotoxicity with the Bartels *Clostridium difficile* cytotoxicity assay kit (Trinity Biotech, Jamestown, NY). The culture fluids were incubated with the fibroblast cells for 24 h and observed under a microscope for cytotoxic effects. Images were taken with an EVOS XL microscope (Advanced Microscopy Group) at ×20 magnification. Panels: A, a representative image of fibroblast cells cultured in growth medium only; B, wild-type 630; C, 630 *agrB1D1* mutant; D, 630 complemented *agrB1D1* mutant; E, wild-type R20291; F, R20291 *agrB1D1* mutant; G, complemented R20291 *agrB1D1* mutant; H, R20291 *agrB2D2* mutant.

