# Supplementary figures and images for: Accessory Gene Regulator-1 Locus Is Essential for Virulence and Pathogenesis of Clostridium difficile
Source: mBio. 2016 Aug 16;7(4):e01237-16. doi: 10.1128/mBio.01237-16 (PMC4992976; doi:10.1128/mBio.01237-16)

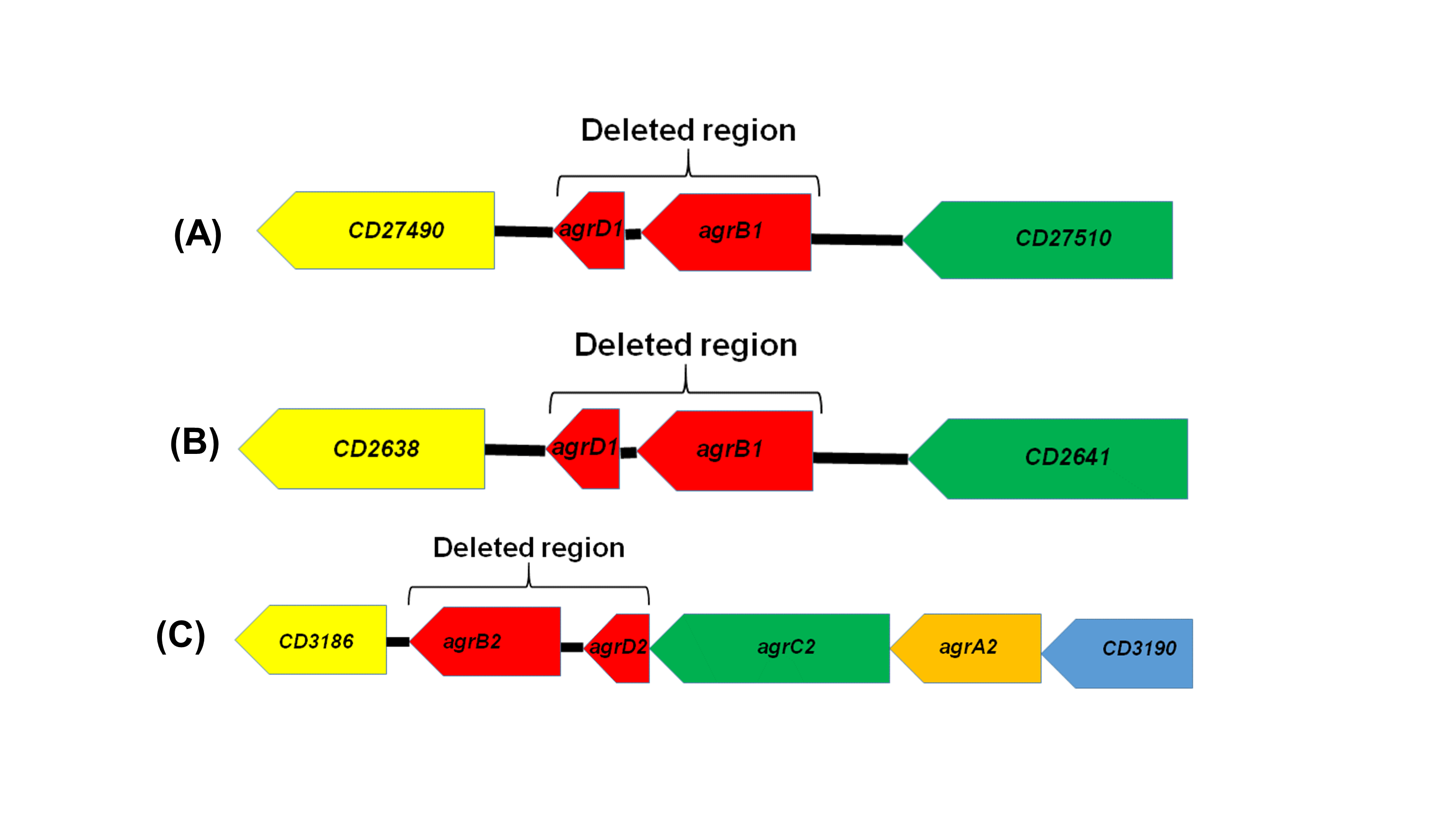

Supplement: Figure S1 — The C. difficile accessory gene regulator loci showing the location of the region deleted by allelic exchange. The agr1 locus in strains 630 (A) and R20291 (B) and the agr2 locus in strain R20291 (C) are shown. Download [file mbo004162940sf1.tif]

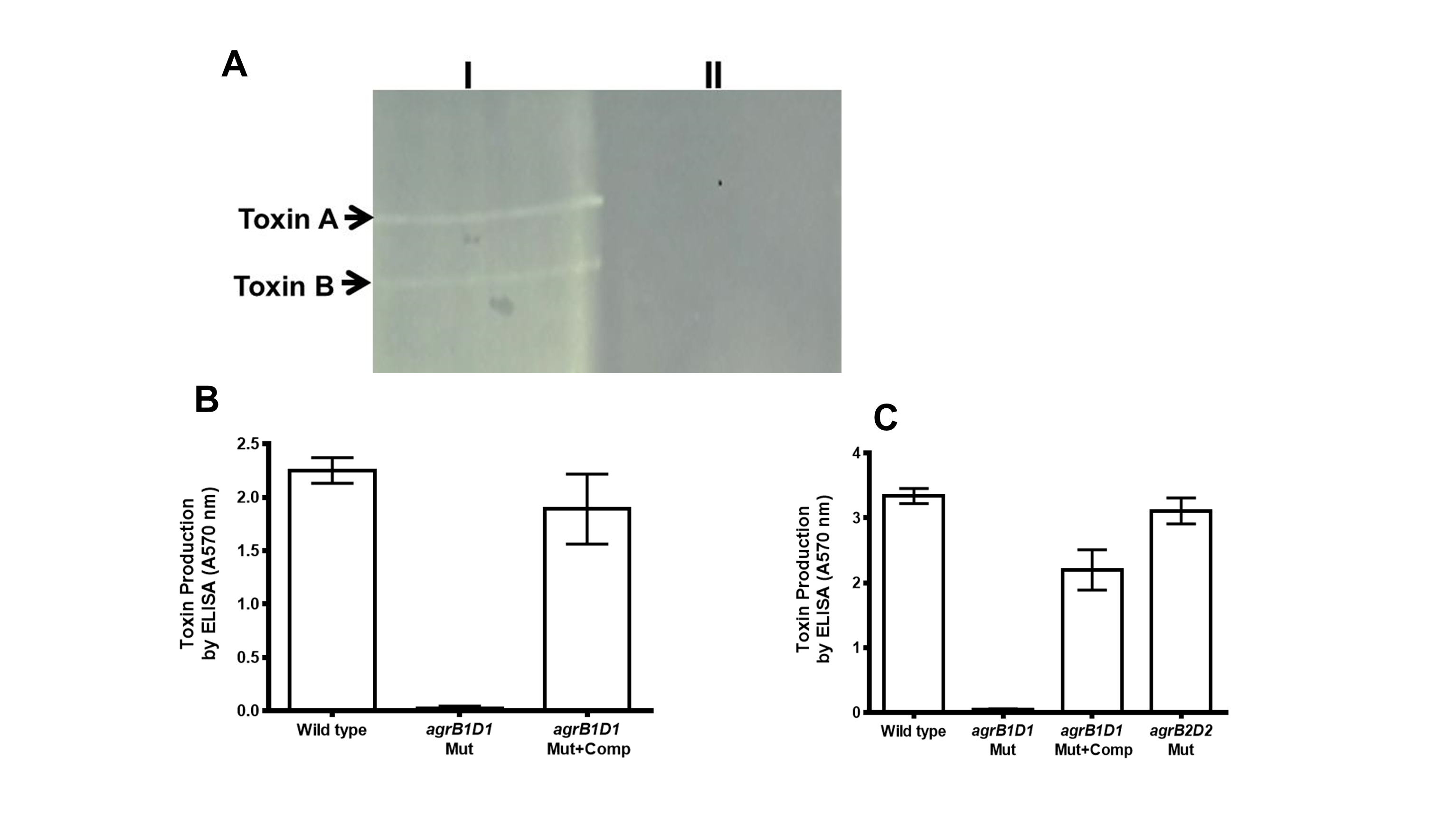

Supplement: Figure S2 — (A) Western blot analysis of 48-h culture supernatant fluid from the 630 agrB1D1 mutant. Supernatant from a 48-h culture was concentrated with the Pierce 150-kDa concentrator (Thermo Fisher Scientific Inc., Rockford, IL) and subjected to 6% PAGE. The protein bands were transferred onto a 0.45-µm nitrocellulose membrane and probed with monoclonal antibodies specific for toxins A and B. The toxin bands on the transferred membrane were detected with the Protein Detector Western blot BCIP/NBT kit (KPL, Gaithersburg, MD). I, wild type; II, agrB1D1 mutant. (B) The agr1 deletion abolishes toxin production in both the 630 (B) and R20291 (C) strains. The agrB1D1 mutants of both strains and a agrB2D2 mutant of the R20291 strain were incubated in BHI medium for 48 h anaerobically at 37°C. Toxin production was tested by ELISA with the Wampole C. difficile TOX A/B II assay (Technologies Lab, Blacksburg, VA). agrB1D1 Mut, agrB1D1 deletion mutant; agrB1D1 Mut+Comp, agrB1D1 mutant complemented with a plasmid bearing the wild-type agrB1D1 locus; agrB2D2 Mut, agrB2D2 deletion mutant. There were significant differences (P = 0.0023 for 630 and 0.0001 for R20291) between the amounts of toxins produced by the wild-type and agrB1D1 mutant strains. Error bars represent the standard deviations of three independent experiments. Download [file mbo004162940sf2.tif]

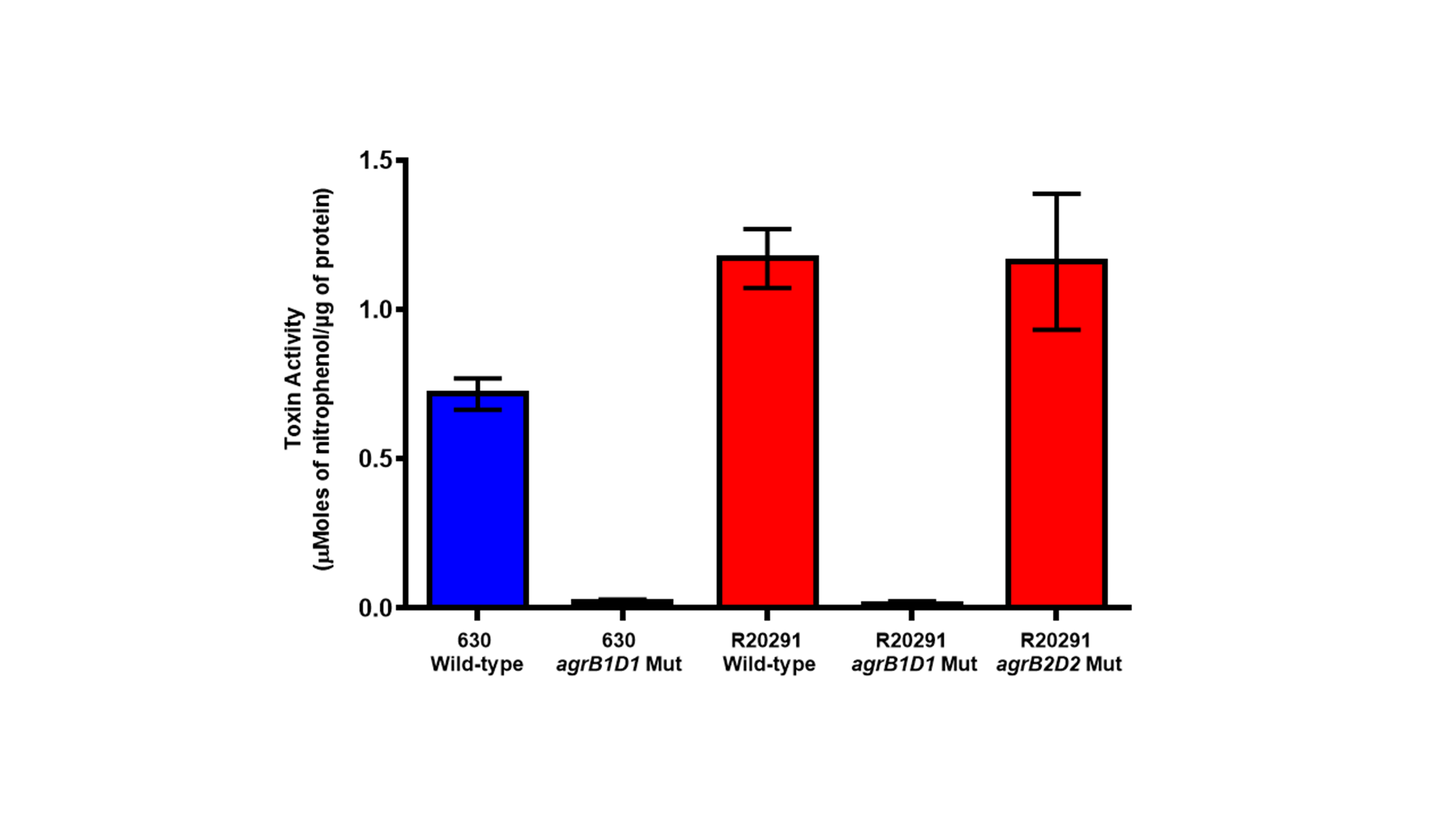

Supplement: Figure S3 — The agr1 mutants do not produce toxin in TY medium. agrB1D1 mutants of both strains and the R20291 agrB2D2 mutant strain were incubated in TY medium for 48 h anaerobically at 37°C. Toxin production was detected with the Cdifftox activity assay. agrB2D2 Mut, R20291 agrB2D2 mutant; agrB1D1 Mut, agrB1D1 mutant. There was a significant difference (P = 0.003 for 630 and 0.0001 for R20291) between the levels of toxin activity produced by the wild-type and agrB1D1 mutant strains. Error bars represent the standard deviations of three independent experiments. Download [file mbo004162940sf3.tif]

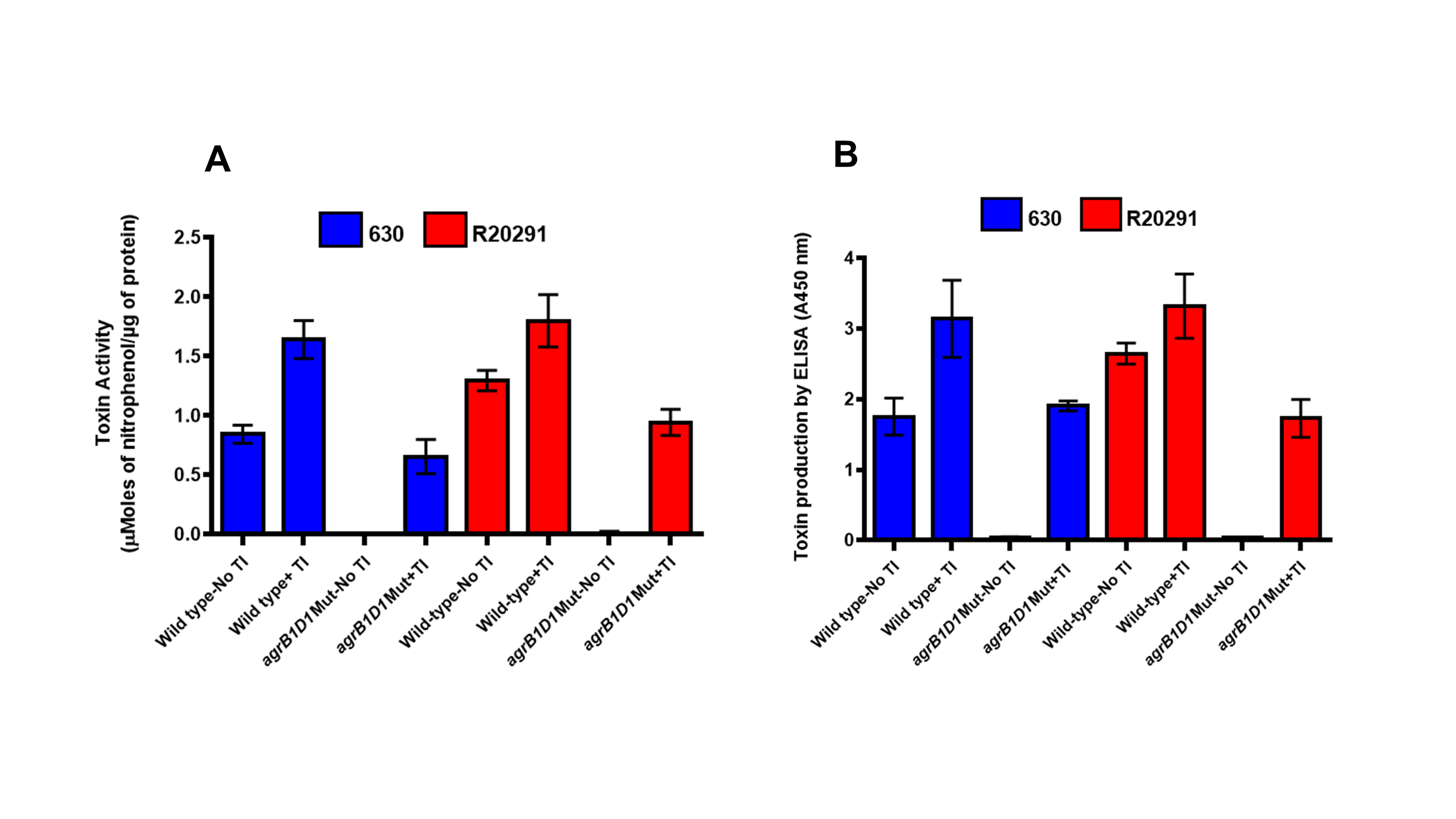

Supplement: Figure S4 — The R20291 agrB2D2 mutant produces an active TI signal that restores toxin production in 630 and R20291 agrB1D1 mutants unable to make toxins. 630 and R20291 agrB1D1 mutant strains were incubated in BHI medium anaerobically for 24 h in the presence of the TI signal purified from the R20291 agrB2D2 mutant. Toxin activity was detected with the Cdifftox activity assay (A), and toxin production was tested by nonquantitative ELISA (B) with the Wampole C. difficile TOX A/B II assay (Technologies Lab, Blacksburg, VA). agrB1D1 Mut, agrB1D1 deletion mutant; TI, TI signal. There were significant differences (P = 0.0041 for 630 and 0.0001 for R20291) between the amounts of toxins produced by the wild-type and agrB1D1 mutant strains in the absence of the TI signal. Error bars represent the standard deviations of three independent experiments. Download [file mbo004162940sf4.tif]

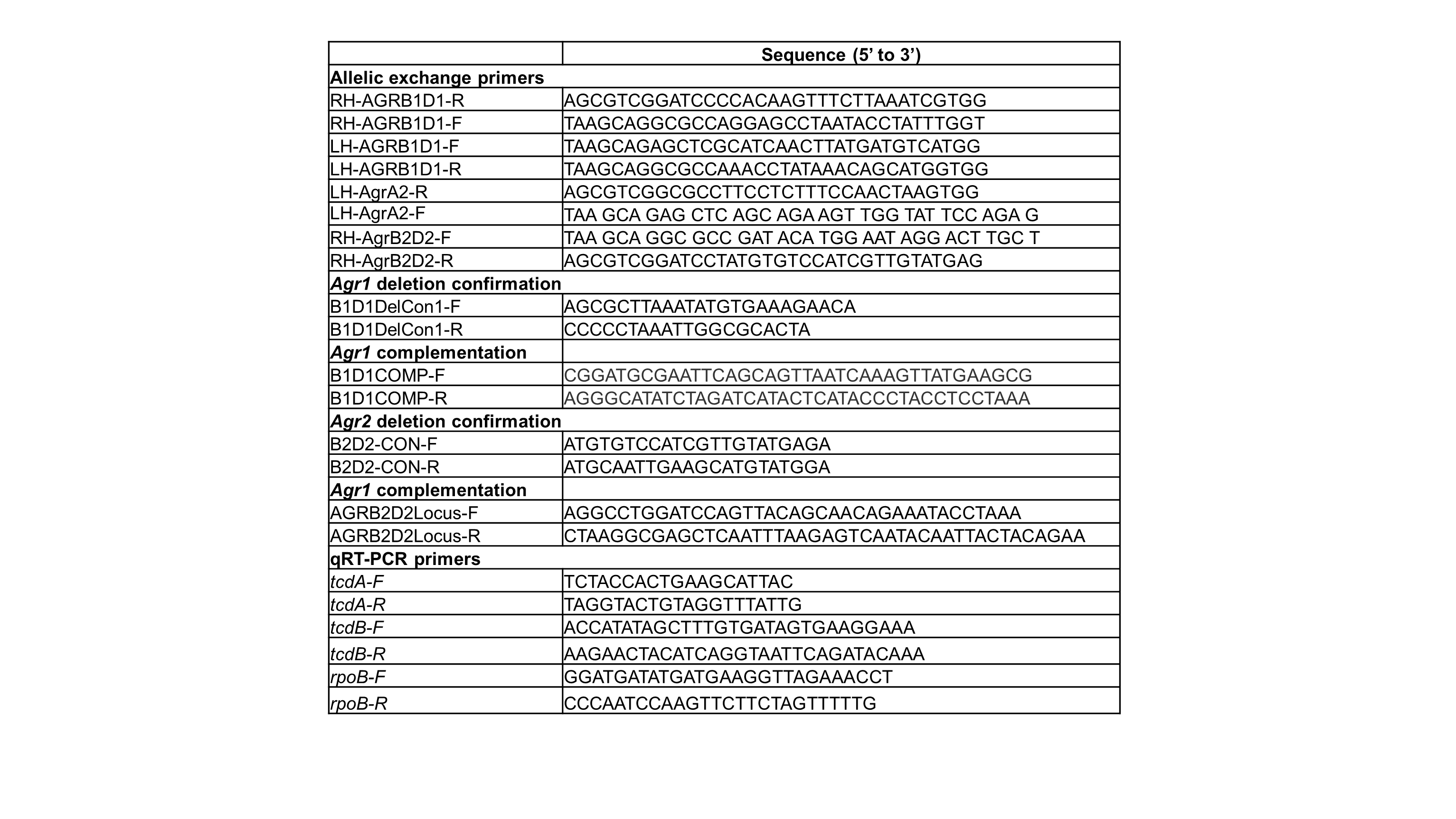

Supplement: Table S1 — Primers used in this study. [file mbo004162940st1.tif]
